# Content-Aware Eye Tracking for Autostereoscopic 3D Display

**DOI:** 10.3390/s20174787

**Published:** 2020-08-25

**Authors:** Dongwoo Kang, Jingu Heo

**Affiliations:** Multimedia Processing Lab, Samsung Advanced Institute of Technology, Suwon 16678, Korea; dkang0309@gmail.com

**Keywords:** eye detection, eye tracking, content-aware eye alignment, error reinforcement learning, autostereoscopic three-dimensional display, augmented reality display

## Abstract

This study develops an eye tracking method for autostereoscopic three-dimensional (3D) display systems for use in various environments. The eye tracking-based autostereoscopic 3D display provides low crosstalk and high-resolution 3D image experience seamlessly without 3D eyeglasses by overcoming the viewing position restriction. However, accurate and fast eye position detection and tracking are still challenging, owing to the various light conditions, camera control, thick eyeglasses, eyeglass sunlight reflection, and limited system resources. This study presents a robust, automated algorithm and relevant systems for accurate and fast detection and tracking of eye pupil centers in 3D with a single visual camera and near-infrared (NIR) light emitting diodes (LEDs). Our proposed eye tracker consists of eye–nose detection, eye–nose shape keypoint alignment, a tracker checker, and tracking with NIR LED on/off control. Eye–nose detection generates facial subregion boxes, including the eyes and nose, which utilize an Error-Based Learning (EBL) method for the selection of the best learnt database (DB). After detection, the eye–nose shape alignment is processed by the Supervised Descent Method (SDM) with Scale-invariant Feature Transform (SIFT). The aligner is content-aware in the sense that corresponding designated aligners are applied based on image content classification, such as the various light conditions and wearing eyeglasses. The conducted experiments on real image DBs yield promising eye detection and tracking outcomes, even in the presence of challenging conditions.

## 1. Introduction

**Figure 1:**
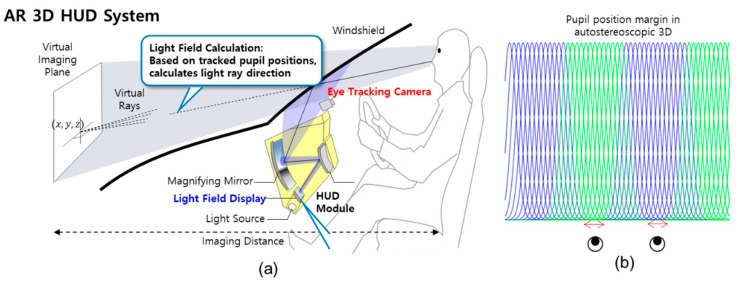
(**a**) Example of an augmented reality three-dimensional head-up display (AR 3D HUD) system and (**b**) autostereoscopic display eye-position margin in the x-direction. A 27-view autostereoscopic 3D display design is shown as an example in (**b**).

Autostereoscopic three-dimensional (3D) displays provide immersive visual experiences with a realistic sense of the image depth without the need for 3D eyeglasses [1,2]. Although multiview autostereoscopic 3D displays can be observed without 3D eyeglasses, they suffer from limited 3D viewing zones and decreased image resolution, owing to the number of 3D views [1,2]. The eye tracking-based autostereoscopic 3D method overcomes these limitations, allows a single-user, seamless 3D experience, and provides higher 3D resolution contents. Several consumer applications for a single user can utilize this technique, such as mobile devices, personal monitors, and game consoles. One of the commercial eye tracking-based 3D display products is the Nintendo 3DS. Additionally, our 10.1” tablet and 31.5” personal monitor prototypes that use the eye tracking-based directional subpixel rendering algorithm are described in [2,3]. Additionally, this eye tracking-based autostereoscopic 3D method can also be utilized in head-up displays (HUDs) (Figure 1a), which have been increasingly used by the automotive industry. Visual contents are shown directly in the line-of-sight via combiners or windshields. While two-dimensional (2D) HUD shows augmented reality (AR) information in a 2D virtual plane that causes additional distraction and visual mismatches, AR 3D HUD can overlap 3D visual information directly on the street or road objects following the adjustment of the 3D depth [4,5]. The real-time eye tracking system can work on various consumer electronics platforms; it is not limited to autostereoscopic 3D display systems including 3D HUD as described. The eye tracking method can be adopted in various consumer electronics, such as driver monitors in automobiles [6,7], augmented and virtual reality [8], smartphones, gaming devices, and televisions [9]. In particular, for driver monitoring systems in automobiles, the eye tracking method can capture the driver’s state by checking the speed of pupil movement and head pose estimation from face normal direction. These can be utilized as indicators of drivers’ attention levels. Further, smartphones and digital cameras can use the eye tracking method for autofocus on human eyes rather than whole faces [10]. Additionally, when the eye-position-tracking method is extended to gaze-tracking techniques when combined with eye glints information, its application extends to a variety of human–machine interactions [11,12,13].

In eye tracking-based autostereoscopic 3D display systems, the left and right images are separated by the 3D eye locations of the viewers, whereby the pixel values are adapted according to the left and right eye positions in real-time by the directional subpixel rendering algorithms [2,3]. With more accurate and faster eye-center position tracking, 3D images with lower crosstalk and higher 3D resolution can be provided, even when the head movements of the users are considered in real-time. The allowed accuracy of the eye tracking position and tracking speed for our real-time 3D system prototypes were described in our previous study [2]. When the optimal viewing distance between the user and the eye tracking camera at vehicles was assumed to be between 400 and 1000 mm considering common drivers’ viewing conditions, the allowed eye-positional error margins were 8 mm in the x-direction (Figure 1b), 20 mm in the y-direction, and 300 mm in the z-direction for the designed 27-view optical design. The overall 3D rendering system latency in our proposed 3D systems consists of the camera capturing, the subpixel rendering, 3D image/graphics rendering, and eye tracking times, whereby the crosstalk increases when the users move [2]. To reduce the crosstalk that is induced when the user’s head moves, faster eye tracking is used which lowers the overall system latency. When the eye tracking fails, it restarts the detection mode, which scans the whole image to find the eye–nose area with relatively lower speed (16 ms), compared to the tracking mode (5 ms) which utilized a small region of interest from the eye tracking success in the previous camera frame. Therefore, it is desirable to maintain the tracking mode at each frame without execution of detection module, in terms of the overall system latency.

**Figure 2:**
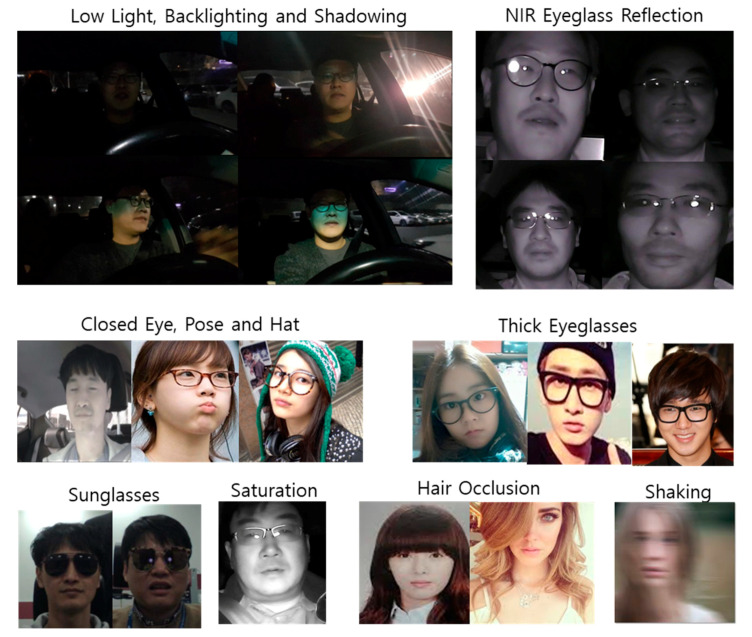
Challenging cases for pupil tracking in real-world applications.

Many efforts have been expended in the development of eye tracking over the last few decades. While earlier approaches were often intrusive and required wearable devices, such as head-mounted devices, recent studies employed eye capturing hardware techniques with computer vision to track eyes at farther distances at approximately 1 m [14]. Reflection from the pupil cornea and bright pupils are commonly used for remote eye-gaze tracking. This scheme requires near infrared (NIR) light sources, whereby the camera sensor and light sources should be located on specific locations each [14,15]. The accuracy of these techniques depends on how well the hardware devices generate and capture eye images. In these techniques, the center positions of the eye are detected by capturing both the bright and dark pupils by adjusting the NIR illuminating sources with long (318 mm) bar-type devices [8]. Additionally, recent advances in computer vision and face recognition algorithms have allowed red–green–blue (RGB) web-camera-based eye position tracking without the need to capture bright or dark pupils or corneal reflections. In these types of methods, pupil centers can be calculated from conventional face detection and facial landmark point-alignment methods [16,17,18,19,20,21,22,23,24,25]. However, fast and accurate face detection and facial shape point alignments are still challenging. Real-time eye detection and tracking are particularly challenging owing to user conditions, such as shaking and pose, various light conditions, system latency, camera control, thick eyeglasses, sunglasses, and limited system resources. NIR light sources are usually adopted in low-light conditions, but they suffer from eyeglass reflection that obstructs eye shape visibility in reconstructed images (Figure 2).

The authors propose a practical and robust computer vision-based, eye tracking algorithm and a relevant system that satisfies autostereoscopic 3D system requirements in various user and environmental conditions. It consists of an eye–nose region detection and an eye–nose facial keypoint alignment and tracking option. The detection algorithms and tracking/alignment are based on machine learning, and not on deep neural networks, whereby only central processing unit (CPU) calculations are required when the limited system resources are considered in real consumer products. The aligner is content-aware in the sense that based on image content classification, such as the various light conditions or instances at which the users wear eyeglasses, corresponding designated aligners are applied. The authors have previously described a preliminary algorithm for the detection and tracking of pupil centers that was validated on simple images [26]. In this study, the authors describe an improved algorithm, with respect to [26], and system targeting real usage on autostereoscopic 3D personal monitors, tablets, and HUD systems. The aim of our study was to develop a robust and practical algorithm to detect and track 3D eye positions from a single camera and simple NIR light-emitting diodes (LED) in various challenging environments. The proposed machine learning-based method was validated on a real-time webcam based on 3D display and a DB that consisted of user face recorded videos.

This study is organized as follows. The proposed algorithm is described in Section 2 and consists of the eye detection and tracking features. In Section 2, our eye tracking system for autostereoscopic 3D displays is also discussed. Experimental results are described in Section 3, and the discussion on the algorithm and results are presented in Section 4. Concluding remarks are outlined in Section 5.

## 2. Materials and Methods

**Figure 3:**
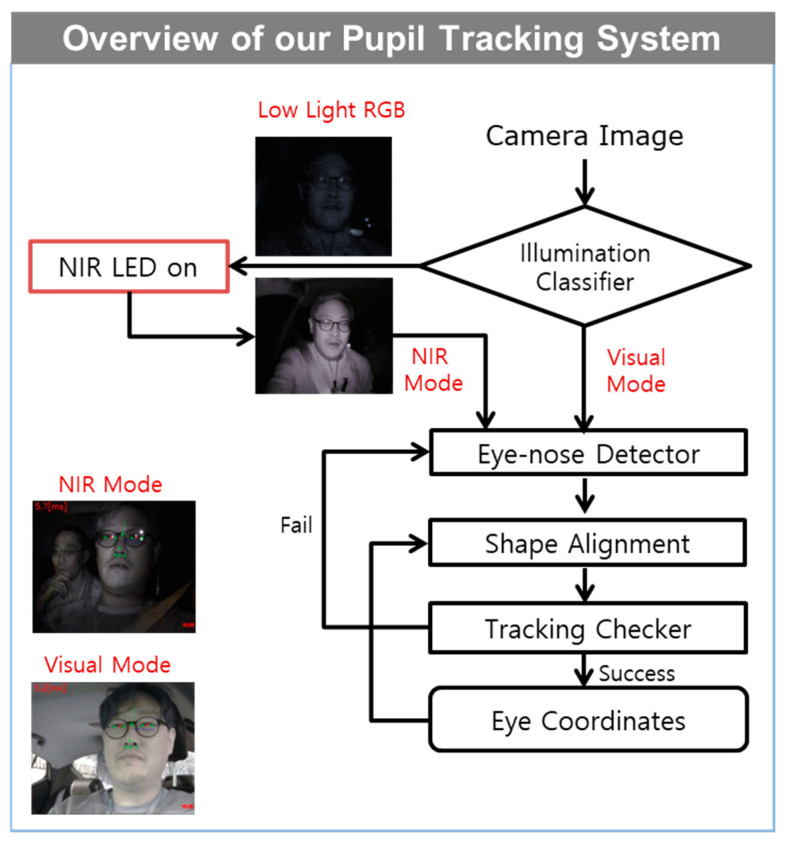
Overview of the proposed eye tracker with near-infrared (NIR) light emitting diodes (on/off). The algorithm consists of an eye–nose detector, eye–nose alignment, and a tracking checker.

The authors categorized the proposed work in three different subsections. In each subsection, first, the authors review related works and explain the proposed work. The basic components of our proposed eye tracker can be divided into two main stages: (1) eye–nose region detection from RGB or NIR webcam images, and (2) position tracking of the eye center from the detected eye–nose region. Once the detection succeeds, the algorithm operates only in the tracking mode until tracking eye position fails. The tracking mode consists of two steps, namely, the eye–nose shape alignment based on 11 landmark points and the tracking of every camera frame with the tracker checker (Figure 3). Note that the tracked 2D eye position is converted to 3D coordinates for autostereoscopic 3D display systems, as described in Section 3.

### 2.1. Eye–Nose Detection

Adaboost is one of the extensively used methods for detecting objects, especially for face detection [18,19,20]. By utilizing intensity differences between patches in an image, known as Haar-like features [18], a strong classifier can be designed by reweighting erroneous samples from weak classifiers under the Adaboost training framework. Owing to the challenging real-world problems for object detection, such as severe pose, extreme low-lighting, obstructions, and low resolution, continuous developments have been made to improve accuracy and stability in this field. Recently, convolutional neural network (CNN)-based face detection approaches, including SqueezeDet [16], region CNN (R-CNN) [21], and multitask CNN (MTCNN) [22], have been developed to overcome some of existing real-world problems such as various light conditions and face partial occlusions, however, they still require computational resources, large corpora of databases for training, expensive training efforts, and considerable graphics processing unit (GPU) power. Therefore, in some applications where fast computation is critical and limited computational resources are available, GPU-based approaches may not be always a desired choice.

To overcome previous object detection approaches for handling real-world problems, the authors propose an Error-Based Learning (EBL) framework. Given that our primary goal is to detect eyes in faces, the authors focused on eye detection instead of face detection or object detection in this study, despite the fact that the main EBL framework could be applied to other detection schemes in the same manner. The basic Adaboost training framework with multiblock local binary pattern (LBP) was used for the basic eye detector. However, previous approaches did not provide guidance on how to train a database with a large size (DB).

The motivation behind EBL is the human learning process. At the child development stage, the human brain establishes neuronal connections continually, while many unused connections or memories are easily eliminated. Based on education, humans continue to develop necessary skills and thoughts that can be suitable for self-survival in society. Through this youth stage, humans continue to update or change their previous ideas and thoughts based on experiences on a trial-and-error basis. As a result, the decision rule may continue to change throughout adulthood, although the number of mistakes humans can make is reduced considerably compared with the childhood and youth stages.

**Figure 4:**
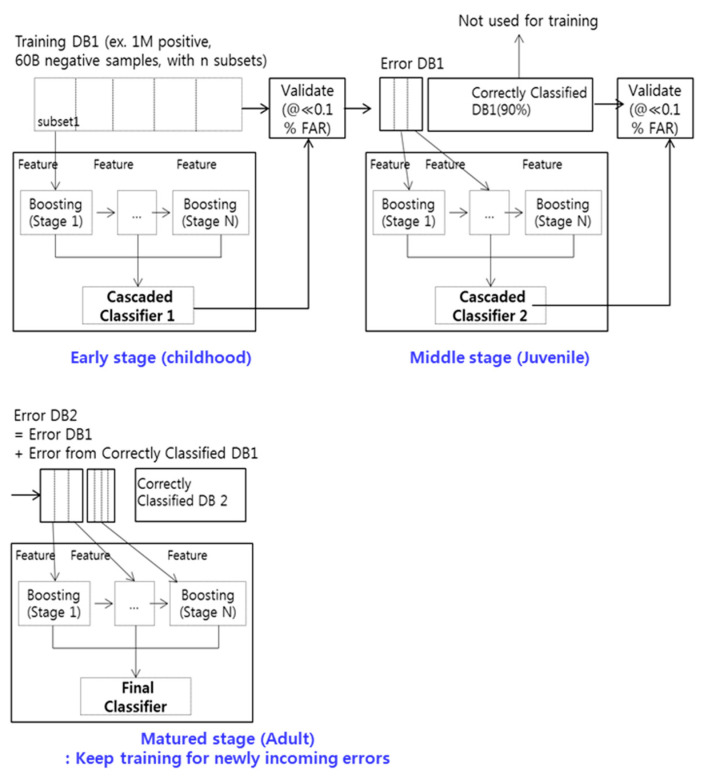
Overview of the Error-Based Learning (EBL) framework.

EBL comprises three stages (Early, Middle, and Mature), which can be analogous to the aforementioned human learning stages. The authors used a cascaded classifier with N boosting substages for each stage in EBL. To handle various user and light conditions, we constructed more than 1 M face (or eye) image datasets which consisted of public DBs such as the CMU Multi-PIE face database [27] and our own captured face images in various circumstances. In addition, various 60 B negative samples for the EBL training were constructed by capturing various scenes without faces. At each training stage of EBL, the number of negative images from 60 B datasets randomly sampled was the same as the number of positive face images for training. At the Early stage, a conventional cascaded Adaboost classifier can be designed by using a subset of the entire training database, 0.1 M images. Given that the total number of training face (or eye) DBs can easily exceed 1 M, the authors did not attempt to start with large corpora for training. Instead, only a subset of the DB, which is randomly sampled, can be used to obtain a cascaded Adaboost classifier at the Early stage. By using the classifier designed in the Early stage, the 1 M entire face images and 60 B negative images were evaluated, and only erroneous samples were collected for both positive and negative samples. Herein, correctly classified samples in the Early stage are removed. In this way, only a small number of important samples for the classification remain, and these erroneous samples are used for the Middle-stage training. Finally, a new collection of erroneous samples, falsely classified by the Middle stage, is also used for training a classifier in the Mature stage. As a result, the final number of erroneous samples classified by the Mature stage was very small relative to the total number related to all the training DBs. Thus, Mature stage training continues to be used if errors exist in the Mature stage. The final classifier after a certain number of iterations of Mature stage training can become much stronger and the training time can be reduced by more than 50 times (5 days versus 3 h with 1 M face images), while accuracy is improved. By utilizing error samples in the Early and Middle stages, the authors searched the new feature space and evaluated all the training samples for the adjustment of the classifier. In the Mature stage, only samples that were important for the classification remained. The authors assumed that these samples were the most important samples for eye–nose classification and should be used for final training. Typically, for eye detection, only 5% of the training samples (50 K out of 1 M samples) were used after the completion of the Mature stage training process. The overall block diagram of the proposed EBL is shown in Figure 4.

**Figure 5:**
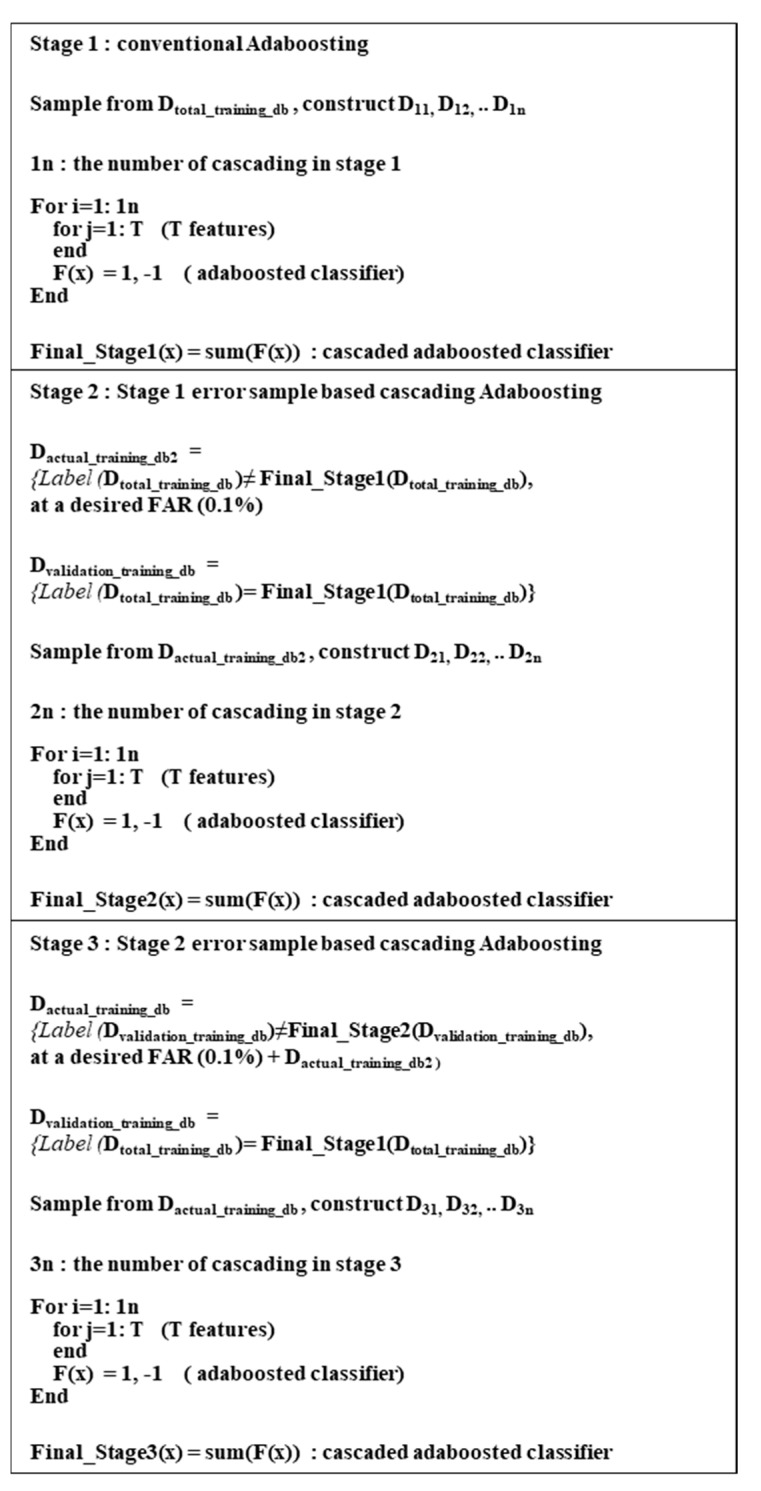
EBL training algorithm using Adaboost: Stage 1 Early stage, Stage 2 middle stage, and Stage 3 final stage.

Suppose the existence of the entire training DB is composed of positive samples, the actual used training DB, and the validation DB in the form of D_total_training_DB_, D_actual_training_DB_, and D_validation_DB_, respectively. Let the corresponding numbers of these databases be N_total_tranining_DB_, N_actual_training_DB_, and N_validation_DB_, and the number of positive samples and negative samples be denoted by N_positive_training_DB_, N_negative_training_DB_, whereby N_negative_training_DB_ >> N_total_training_DB_. The procedures used for training EBL can be explained in Figure 5.

### 2.2. Content-Aware Eye Alignment

After the detector identifies the eye–nose region, the tracking mode starts to extract the coordinates of the centers of the pupils based on eye–nose shape alignments with the use of the supervised descent method (SDM) [17], and the scale-invariant feature transform (SIFT) [23]. With the extracted SIFT features from the eye–nose image region, regression-based landmark point alignment is performed by SDM, whereby the SDM model trains a sequence of descent directions that minimize the mean of the nonlinear square functions from each landmark point [23]. Compared with the conventional shape modeling techniques, such as the active appearance model (AAM) [24], and active shape modeling (ASM) [25], the SDM does not train any shape or appearance model. This overcomes the problem of the excessive computational cost and yields improved accuracy. Recent advances in deep learning techniques yielded CNN-based facial point alignment methods, such as MTCNN [22]. However, these CNN-based methods still require considerable system resources—such as GPU and expensive training efforts—that are associated with speed issues in real-time HUD systems.

**Figure 6:**
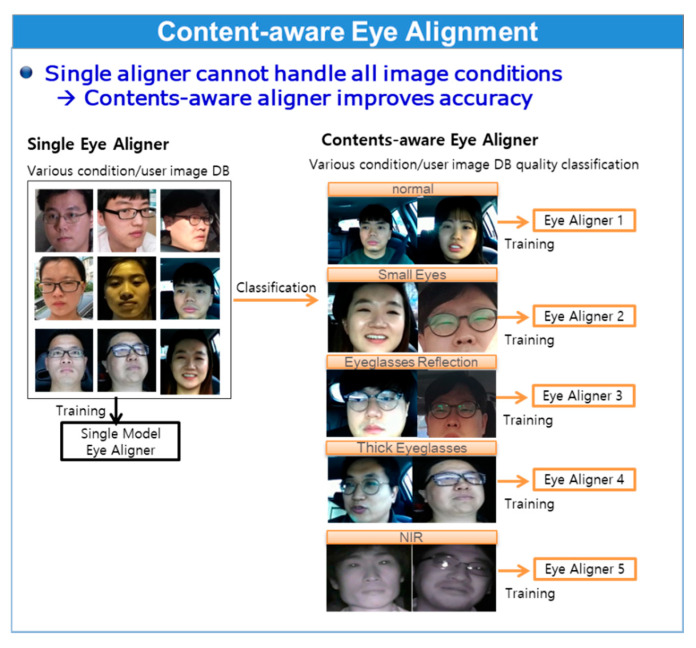
Single aligner model (**left**) versus content-aware quality specific aligner model (**right**).

Because SDM, which is a regression-based method, solves the shape alignment problem as a general optimization problem, the single SDM aligner cannot be easily optimized globally on the training image DB in various conditions. To handle this problem, the authors propose a content-aware eye–nose shape alignment method. The proposed method is content-aware in the sense that different SDM aligners are applied according to the image content categories. Content classification was performed on the twice enlarged eye–nose region obtained from eye–nose alignment in the previous camera frame, and each of the DB performed its own SDM aligner model training and testing. Based on the image quality and condition, such as bright or dark images, NIR images, eyeglass reflections, use of thick eyeglasses, etc., corresponding aligners were applied. This step is illustrated in Figure 6. Unlike EBL-based detector model training, each content-aware SDM aligner trains the content of each image dataset without utilizing error samples, based only on error-free samples to achieve better representation of the image content characteristics.

### 2.3. Tracker Checker

To prevent erroneous detection or tracking, the authors propose a novel tracker checker idea. The proposed tracker checker guarantees that the aligned results contain the eyes. Once the eye-nose shape alignment process is executed, the proposed tracker checker performs the final examination of the tracking results irrespective of whether it tracks eyes or not. Small eye-nose regions calculated from the tracked eye-nose alignment are used for machine learning-based classification as tracker checkers. If the aligned results are judged poorly by the tracker checker, the tracking mode stops, and the eye-nose detector restarts at the subsequent frame. Otherwise, the eye tracking system maintains the tracking mode by executing the eye-detector module. In this way, more efficient and faster eye tracking system computations can be achieved.

**Figure 7:**
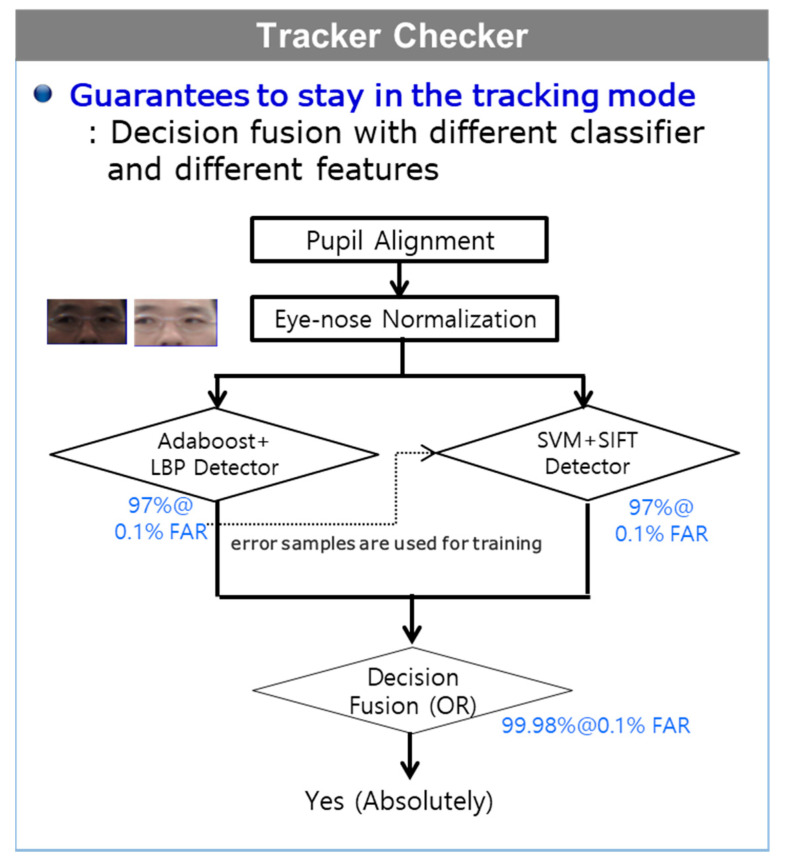
Tracker Checker overview.

Our proposed tracker checker consisted of SIFT features and support vector machine (SVM) classifiers [28], along with the LBP-based eye detector that was used in the eye detector under the EBL framework. With the use of two different feature spaces, such as SIFT and LBP, and with the use of two different classification schemes, such as a set of weak classifiers (Adaboost) and a strong classifier (SVM), our designed tracker checker is guaranteed to be maintained active as long as the aligned results contain the two pupils (Figure 7). Therefore, most of the time during which the user is present in front of our eye tracking system, the tracker checker tries to maintain the tracking mode instead of the detection mode.

### 2.4. Eye Tracking Systems for Autostereoscopic 3D Displays

Based on each component, as described in Section 2, the authors have designed an overall 2D eye tracking scheme for autostereoscopic 3D displays. The 3D position of the pupil centers is calculated along a direction normal to the face and at a fixed interpupillary distance (IPD). The direction normal to the face is estimated based on the alignment of the 3D face model to the 2D tracked 11 eye–nose landmark points. The authors utilized a widely used 3D face model, the Candide-3 head model [29], which is a generic 3D face model that consists of 113 vertices and 168 surfaces. This facial model deforms according to the change in positions and movements of mouth, nose, eyes, etc. [30]. The IPD was assumed to have a fixed value of 65 mm, which is a median value of adult IPDs in the range 50 and 75 mm [31]. Using a fixed IPD is a limitation of our study.

**Figure 8:**
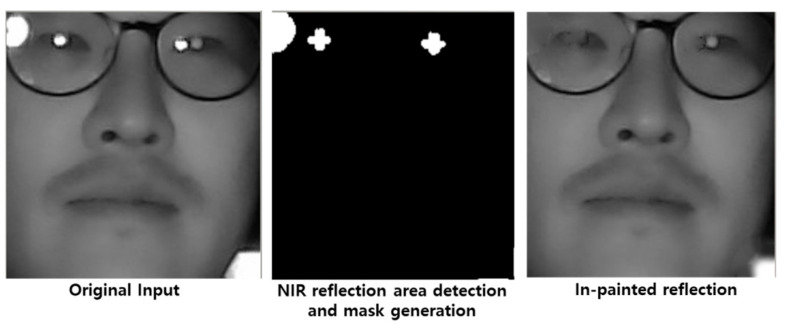
NIR eyeglass reflection detection and removal by an in-painting algorithm.

The overall flowchart of the proposed system and algorithm is shown in Figure 3. One of the noticeable challenging problems that degrade the performance of the eye tracking system is low lighting. Given that most of the high-speed camera sensors do not properly capture images in low-light conditions with a high speed (need at least 60 fps for latency issues), the authors utilized an NIR camera (with active LED lighting), which was turned on when the visual image quality was extremely poor in low-light conditions. Illumination classification was performed to switch the visual/IR camera. Herein, the authors removed the R cut filter, and only a single camera was operated for both the visual and NIR modes of operation. Basic components, as discussed in Section 2, that is, the eye–nose detector, eye–nose alignment, and pupil tracking, were designed for each modality with corresponding quality measures to handle a wide range of real-world image conditions. For each modality, image content-based tracking modules were operated. In other words, by using the visual aligner and the NIR aligner in Figure 6, our eye tracking system could overcome a wide range of image/illumination/user conditions by switching between the visual/NIR modes in a smart manner. Specifically, an in-painting algorithm was applied on an eyeglass reflection area of the NIR DB images for both training and testing as a preprocessing stage for the aligners [32]. Because our eye-alignment algorithm utilized pixel gradient information, the authors in-painted the reflection area in a way that minimized the edges from NIR reflections (Figure 8). This was a necessary step used to improve robustness in the instances at which eyeglasses were present in NIR conditions. It is worth mentioning that the use of the eye–nose region in Section 2 focused more on pupil center alignments, and thus, prevented pupil centers from erroneous alignments, which were less affected by the alignment of other parts of the face in various illumination conditions.

Pixel-to-pixel Euclidean distance mean error normalized by the IPD was applied to evaluate the performance for the proposed eye aligner precision. We also used cumulative error analysis on the normalized mean error.

## 3. Results

**Figure 9:**
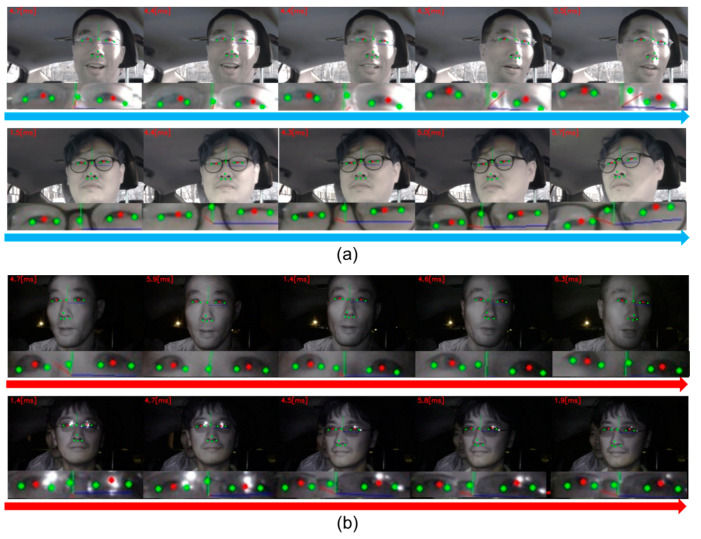
Seamless pupil tracking while driving outside with normal red–green–blue (RGB) (**a**) and NIR (**b**) cameras. The eye tracking speeds are shown at the left upper positions of each frame image such as 4.7 [ms].

The proposed algorithm was implemented with C++ and yielded successful real-time detection (~60 fps) and tracking (~200 fps) with different environments, users, and system challenges, based only on CPU computations. When the tracking mode was considered, the execution time was approximately 5 ms on a standard 2.5 GHz personal computer which ran Windows 7. Additionally, when tested on a commercial mobile tablet, a commercial embedded computing board for 3D HUD achieved almost the same eye tracking speed. Figure 9 shows some of our real-time seamless pupil tracking examples during actual driving outdoors in normal (RGB camera in Figure 9a) and low-light conditions (NIR camera in Figure 9b) with a single camera with NIR LEDs. The camera image resolution was 640 × 480 with a capturing speed of 60 fps, a 60° × 40° field-of-view, and an NIR LED emission wavelength of 940 nm. This worked robustly in illumination changing conditions with or without eyeglasses while the head was moving.

The detector model was trained with eye–nose region labeling on, with various RGB and NIR face image datasets with the use of the EBL method. The training samples at the Mature stage of the EBL method included 660,000 RGB and 60,000 NIR image datasets. For the SDM aligner, different models were trained separately with 11 eye–nose landmark points as the ground truth on different image content categories. The authors generated several different aligner models for our content-aware eye trackers: good quality RGB images in normal light conditions, moderate quality RGB images in bright and dark light conditions, poor quality RGB images with thick eyeglasses, good quality NIR images, and bad quality NIR images with eyeglass reflection (Table 1). For each aligner model, the authors trained with 60,000 image training samples. The proposed SVM-based tracker checker used the same image DB as that used for each different SDM aligner.

The authors tested our algorithm on image and video DB entries captured in normal office environments (100–400 lux) and in challenging conditions outdoors based on images of drivers or passengers during the daytime, sunset, and night times (5–10,000 lux). For the image DB evaluation, 100,000 conditioned RGB images and 30,000 NIR images were tested, which were not used in the training process. Additionally, short-video datasets (approximately 30 s each, with a capturing speed of 60 fps at distances of approximately 0.6 m from the camera) were recorded inside the office based on adjustments of the light conditions and outside during actual driving in the morning, afternoon, and night times. The authors tested 10 RGB and 10 NIR videos of various people. The average detector accuracy was 99.4% on the RGB image DB and 98.1% on the NIR image DB. The average aligner precision error was 1 ± 0.7 mm on the RGB images and 2 ± 1.1 mm on the NIR images (Table 2). The aligner precision measurement was calculated by the distances between the ground truth and the tracked pupil centers, whereby the pixel distances were converted to physical distances based on the assumption that the IPD was 65 mm. For the video dataset, the authors obtained a detector accuracy of 99.1% on the RGB and 99.9% on the NIR, and an aligner precision of 1.5 ± 0.9 mm on the RGB and 2.0 ± 1.2 mm on the NIR. The overall performance of the proposed eye tracker had a detector accuracy of 98.7%, a tracker precision error of 1.5 ± 0.8 mm, and a tracker checker accuracy of 99.9%. The cumulative mean errors were 83% under 1 mm error, 92% under error 2 mm error, 98% under error 3mm error, and 99% under 5mm error.

## 4. Discussion

Our results demonstrated high accuracy and fast speed regarding the tracking of the position of the eye center in various illumination and user conditions. The algorithm was validated on image and video datasets in indoor environments in which light was adjusted, and in actual driving conditions outdoors that targeted real consumer products such as personal monitors, tablets at home and offices, and HUD in vehicles. The authors defined normal conditions as indoor normal light conditions in offices (100–400 lux) without any obstacles for the alignment of eye shapes, including cases in which the subjects wore thick eyeglasses. When the performances in normal conditions (99% at 1 mm) and challenging conditions (98% at 2 mm) were compared, both the detector and the aligner performances diminished in the cases of challenging conditions. Table 3 shows the detailed performance of the proposed method in various challenging conditions. In the RGB mode of operation, eye tracking datasets pertaining to sunlight reflection on eyeglasses yielded the largest alignment errors (4.3 mm).

**Figure 10:**
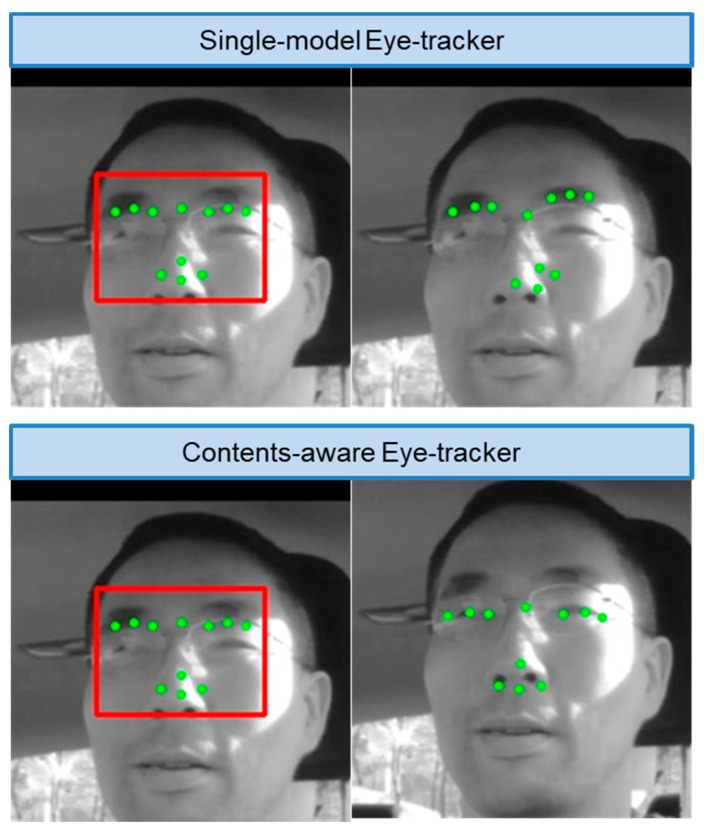
Single-model eye-tracker versus the proposed content-aware eye-tracker on one of the challenging cases that related to eyeglass reflection based on sunlight. The initial shape points inside the detector box (**left**) are regressed to optimal shape points by the content-designated, SDM-trained aligner (**right**). Compared with the conventional single-model eye-tracker, the proposed content-aware eye-tracker successfully aligned the correct eye positions.

Specifically, wearing eyeglasses without antiglare coatings caused inaccurate tracking (Figure 10). For NIR modes, the detector performance was almost the same for each challenging case, but the aligner suffered from eyeglass NIR reflection with large errors (2.9 mm). This indicates that our SDM-based aligner performance depends on how pupils are clearly captured by cameras. When the average pupil size (radius = 3 mm) was considered in normal light conditions, the results generated for challenging cases were still reasonable.

One of the main advances compared with previously published works is that our method was content-aware, whereby different eye trackers were applied according to the image conditions, such as normal, bright, dark, low-light cases, and cases with thick eyeglasses and eyeglass sunlight reflection (Figure 10). Specifically, low-light conditions can be handled with NIR LED control and with the NIR image DB-based eye tracker. Additionally, compared with previous methods that detected faces or eye–nose regions, our proposed detection utilized the novel EBL approach. The EBL method typically trains only a small fraction (less than 5%) of the detection training image DBs in much shorter training times, while improving the detection rate through three stages.

A number of studies attempted automatic face detection and facial landmark point alignment with higher accuracy, including deep learning-based techniques. Despite the fact that even the state-of-the-art, deep learning-based methods accomplish considerable improvements compared with classical methods, they suffer from increased computational resources and lower speed requirements. This study proposed a practical eye-center position-tracking method, whereby our method was based on face detection and alignment techniques, but with priority assigned to the accuracy of the eye position. To minimize the errors from other shape misalignments, we reduced the number of points to 11 (i.e., two eye center points, four eye shapes, and six nose shape points after extensive experiments on different points). The performance comparison between our proposed algorithm and one of the fastest deep learning detector techniques, SqueezeDet (57.2 fps) [16], is listed in Table 4. Additionally, our proposed tracker was compared with one of the most precise deep neural net-based eye–face alignment methods, MTCNN (Table 4) [22]. While CNN-based methods require considerable GPU computations, our content-aware pupil tracking algorithm achieved higher detection accuracy, lower pupil precision error, and higher speed in both the high- and low-light conditions with CPU computation.

To validate the effectiveness of our method, the authors carried out a 3D crosstalk study with our autostereoscopic 3D display prototype systems. The total system latency was approximately 70 ms, which included the camera capturing time, eye tracking time, 3D and graphics rendering time, data transmission time, and display output time. Subjective 3D quality tests of the autostereoscopic 3D display were also performed with the proposed eye tracking method. Users did not experience any crosstalk with our eye tracker when they moved at normal speeds, but they experienced increased crosstalk during fast head movements (>0.2 m/s) owing to the system latency. Specifically, when the eye tracker restarted from the detector mode after tracking was lost, the users experienced distinct crosstalk. This can occur when side mirrors or side cars are checked while driving, whereby the user’s head pose is outside the allowed pose limit (yaw, raw, pitch of 30°) of our eye-tracker algorithms.

There are a few limitations associated with our study. Our proposed method suffers in the cases at which the pupil’s shape is obstructed, such as the cases in which subjects wear sunglasses, or when sunlight is reflected on eyeglasses. Specifically, NIR eyeglass reflection decreased the precision and accuracy of the tracking of the pupil center. NIR reflection can be minimized by adding a special hardware system solution, such as the separating camera sensor, NIR LEDs, and dynamic NIR LED light control. Additionally, wearing sunglasses yielded limited performance in eye alignments because pupil locations were estimated with other shapes, owing to the invisibility of the pupils due to the sunglasses. To increase the instances in which sunglasses can be used, personalized pupil tracking technologies will be studied in the future.

**Figure 11:**
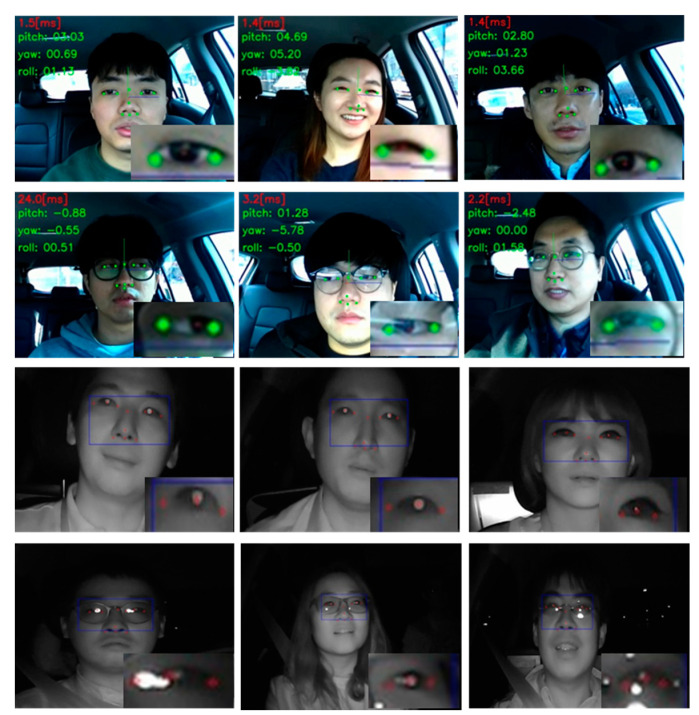
Additional results at various driving conditions: RGB mode at daytime (1st and 2nd rows) and NIR mode at nighttime (3rd and 4th rows). Especially, the 1st and 2nd rows show head pose estimation results from our 11 point eye tracking algorithm.

Figure 11 displays an additional number of results of our test faces acquired during actual vehicular driving.

## 5. Conclusions

This study presented a practical real-time pupil tracking system and an algorithm that used a single camera and a NIR LED. Our proposed method yielded robust performance in various illumination conditions, which is extremely important and highly beneficial for AR 3D HUD. This demonstrates the proposed method’s superior performance compared with other state-of-the-art approaches for pupil tracking in terms of accuracy and speed, and with the use of fewer computational resources.

## Figures and Tables

**Table 1:** Training DB for detector and Aligner.

**Training DB (Detector)**	**DB Type**	**DB Number**
joint DB (RGB + NIR)	RGB DB (general illumination)	660,000
	NIR DB (low illumination)	60,000
**Training DB (Aligner)**	**DB Type**	**DB Number**
content-aware DB (separated DB training)	normal (RGB)	30,000
	small eyes (RGB)	30,000
	eyeglass reflection (RGB)	30,000
	thick eyeglasses (RGB)	30,000
	normal (RGB)	30,000
	eyeglasses reflection (NIR)	30,000

**Table 2:** Testing DB for Detector and Aligner.

Testing DB	DB Type	Detection Accuracy	Tracker Precision
general illumination	office area (100–400 lux)	99.8%	1 mm
	driving outside (50–10,000 lux)	99%	2 mm
low illumination	driving outside (5–50 lux)	98.1%	2 mm

**Table 3:** Performance in various challenging conditions.

Testing DB	Detection Accuracy	Tracker Precision
average normal (RGB)	99.8%	1 mm
average driving outside (RGB)	99%	2 mm
small eyes (RGB)	100%	3.1 mm
eyeglass reflection (RGB)	99.7%	4.3 mm
thick eyeglasses (RGB)	93.8%	1.9 mm
average low light (NIR)	100%	2 mm
wearing eyeglasses in low-light conditions (NIR)	99.1%	2.9 mm

**Table 4:** Performance comparison between previous studies and the proposed algorithm.

**Testing DB (Detector)**	**SqueezeDet [16]**	**Proposed**
normal light (RGB)	99%	99.4%
low light (NIR)	35.1%	98.1%
Eye-glasses (DB)	89.31%	99%
speed	57.2 fps (GPU)	60 fps (CPU)
**Testing DB (Aligner)**	**MTCNN [22]**	**Proposed**
normal light (RGB)	4.2 mm	1 mm
low light (NIR)	5.4 mm	2 mm
eye-glasses (DB)	6.7 mm	2 mm
speed	16 fps (CPU)	200 fps (CPU)
	99 fps (GPU)	
